# Assessing microplastic contamination in milk and dairy products

**DOI:** 10.1038/s41538-025-00506-8

**Published:** 2025-07-10

**Authors:** E. Visentin, G. Niero, F. Benetti, C. O’Donnell, M. De Marchi

**Affiliations:** 1 Department of Agronomy, Food, Natural resources, Animals and Environment, University of Padova, Legnaro, Italy; 2 European Center for the Sustainable Impact of Nanotechnology, EcamRicert S.r.l, Padova, Italy; 3 School of Biosystems and Food Engineering, University College Dublin, Belfield, Ireland

**Keywords:** Polymer characterization, Characterization and analytical techniques, Environmental impact

## Abstract

The presence of microplastics in food has raised growing concern due to potential health risks. While many studies have investigated microplastics in water and seafood, limited data are available for dairy products. This study qualitatively and quantitatively characterizes microplastics in milk, fresh cheese, and ripened cheese, assessing concentration levels and polymer composition through the analysis of 28 dairy samples using Fourier-transformed infrared micro-spectroscopy in attenuated total reflectance mode. Poly(ethylene terephthalate) was the most frequent, followed by polyethylene and polypropylene. Most microplastics were smaller than 150 μm, with 51–100 μm being the most common (33.8%). Irregular fragments (77.4%) and grey particles (68.4%) were predominant. Ripened cheese exhibited the highest microplastic concentration (1857 MP/kg), followed by fresh cheese (1280 MP/kg) and milk (350.0 MP/kg). Results confirm widespread microplastic contamination in dairy products and highlight the importance of further research into contamination pathways and strategies to reduce microplastic exposure in the dairy chain.

## Introduction

The presence of microplastics (MP) in the environment has become a growing concern due to their widespread distribution, persistence, and potential adverse effects on human health. Microplastics are defined as plastic fragments ranging in size from 0.1 µm to 5,000 µm^1,2^. Microplastics have been detected in various natural environments, including oceans^3^, inland waters^4,5^, sediments^6,7^, and biota^8,9^. The accumulation of MP in natural environments raises urgent concerns about its long-term impacts^10^. Due to their small size, MP can accumulate through different trophic levels and enter the food chain, with potential impacts on both ecological systems and human health^11^.

The dietary pathway represents the main route through which MP enters the human body^12–14^. Indeed, MP have been detected in various food categories, including drinking water^15–17^, fish and seafood products^18,19^, honey^20,21^, table salt^22–24^, beer^25–27^, and beverages^28^. The ingestion of MP through contaminated food raises important questions about their bioavailability, accumulation, and potential health risks, especially from a long-term exposure perspective^29^.

Dairy products have received limited attention in respect to the characterization of their potential MP content. Products such as powdered milk, liquid milk, cheese, and yogurt may be contaminated with MP at various stages along the supply chain^30,31^. Contamination may occur at the farm level, through contaminated feed^32^, milking equipment, or clothing^20^. Contamination may also happen at the plant level, during processing, again arising from clothing and protective gear, including hairnets, disposable laboratory coats, and gloves^20,33^. Moreover, contamination can also arise from food additives containing MP residues^34^, as well as from processing machinery, transportation, and storage^35^. Given the complexity of the dairy sector and the extensive use of plastic materials along the entire production chain, understanding the pathways through which MP enter dairy products is crucial for ensuring food safety and assessing potential health risks^30^.

Existing MP studies have focused on human breast milk^36,37^, powdered milk^33,38,39^, raw milk^33,40^, and packaged drinking milk^20,33,41–43^, probably due to their relatively simple detection in terms of analytical procedures, with particular regard to the digestion and filtration steps required for samples^44,45^. However, only a limited number of studies have investigated MP contamination in dairy products. Rbaibi Zipak^46^ assessed the presence of MP in yogurt during individual production steps. Similarly, Buyukunal^47^ investigated MP presence along the production of milk-based beverages, detecting MP in all examined samples. Additionally, Banica^48^ analyzed high-fat dairy products, including butter and sour cream samples, reporting significant contamination also in these products. The presence of MP in such products highlights the need for comprehensive studies to assess contamination levels and potential health risks associated with MP ingestion through various dairy products.

This specific research gap highlights the need for further investigation into the contamination of MP in dairy products. Thus, this study aims (i) to qualitatively and quantitatively characterize MP contamination in milk, fresh cheese, and ripened cheese, and (ii) to assess differences in contamination levels across these different products.

## Results and discussion

### Characterization of MP in dairy products: size, shape, and color

**Fig. 1:**
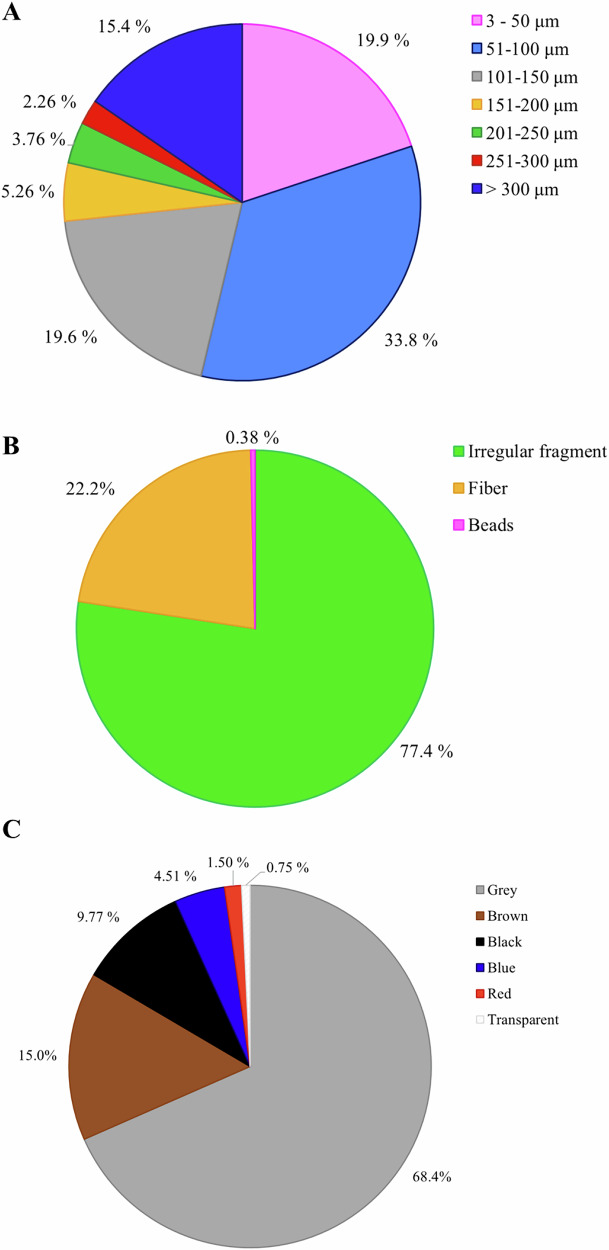
Qualitative characteristics of MP in dairy products. Frequencies (%) of size classes (**A**), shape (**B**), and color (**C**) of MP identified in dairy products.

Descriptive statistics for sizes of MP detected in dairy products are presented in Table 1. Sizes ranged from a minimum of 24.0 μm to a maximum of 4817 μm, with an overall average size of 243.7 μm. Among polymers, polyacrylate exhibited the largest average size (1048 μm), whereas polyvinylidene fluoride and ethylene vinyl acetate had the smallest (32.0 μm and 37.5 μm, respectively). As the most frequently detected MP, poly(ethylene terephthalate), polypropylene, and polyethylene had average sizes of 775.8, 114.4, and 109.0 μm, respectively, with relatively high coefficients of variation (112.5, 127.8, and 55.2%, respectively). As shown in Fig. 1A, 33.8% of the identified MP had sizes comprised between 51 μm and 100 μm, followed by MP in the range between 3 μm and 50 μm (19.9%), and by MP in the range between 101 μm and 150 μm (19.6%). Larger MP fragments, ranging from 201 μm to 250 μm and from 251 μm to 300 μm, were less frequent, representing 3.76% and 2.26% of the total, respectively. The predominance of small MP is consistent with previous studies that reported a similar size distribution, with the majority of particles measuring below 150 μm^46,47^. In a study on MP contamination during the yogurt production process, Rbaibi Zipak^46^ reported that 43.3% of the detected MP had sizes between 1 μm and 150 μm. Buyukunal^47^ analyzed MP contamination along the production of milk-based beverages and reported that 37.4% of detected MP were also in the 1–150 μm range. The similarity between our results and those reported for yogurt and milk-based beverages suggests that MP contamination mechanisms in dairy products are consistent, even across different production processes. The predominance of MP below 150 μm suggests contamination from processing equipment, plastic packaging degradation, or the deposition of airborne particles during production^49^.

**Table 1:** Descriptive statistics for sizes (μm) of MP detected in dairy products

MP	Detected MP, *n*	Mean	SD	CV, %	Minimum	Maximum
Chlorinated polyethylene	5	87.6	28.4	32.4	51.0	124.0
Copolymers^a^	16	79.4	52.4	66.0	31.0	212.0
Ethylene vinyl acetate	2	37.5	12.0	32.1	29.0	46.0
Nylon	5	138.8	124.6	89.8	42.0	352.0
Polyacrylate	8	1,048	1,561	149.0	73.0	4,817
Polybutadiene	1	40.0	–	–	–	–
Polyester	4	68.3	27.3	40.0	42.0	106.0
Polyethylene	76	109.0	60.2	55.2	35.0	325.0
Poly(ethylene terephthalate)	47	775.8	873.1	112.5	41.0	3,766
Polyisoprene	5	60.0	17.3	28.9	40.0	81.0
Polyoxymethylene	1	40.0	–	–	–	–
Polyphenylene sulfide	1	95.0	–	–	–	–
Polypropylene	50	114.4	146.2	127.8	24.0	903.0
Polystyrene	18	77.4	36.1	46.6	34.0	143.0
Polytetrafluoroethylene	5	98.0	40.8	41.6	31.0	131.0
Polyurethane	2	86.0	76.4	88.8	32.0	140.0
Polyvinyl chloride	6	78.2	36.6	46.8	38.0	141.0
Polyvinylidene fluoride	1	32.0	–	–	–	–
Resin	1	70.0	–	–	–	–
Silicone	12	91.9	34.0	37.0	40.0	145.0

^a^Copolymers class comprises: acrylonitrile–butadiene–styrene, chlorinated polyethylene–polystyrene copolymer, polyacrylonitrile–butadiene–styrene copolymer, polyethylene–nylon copolymer, polyethylacrylate–polystyrene–polyacrylamide copolymer, polystyrene–polyacrylate copolymer, polystyrene–polybutadiene copolymer, silicone–polyisoprene copolymer, and styrene–ethylene–butylene–styrene.

*SD* standard deviation, *CV* coefficient of variation.

Data regarding the shapes of detected MP are presented in Table 2 and Fig. 1B. Beads were found exclusively within the copolymers class, representing for 0.38% of the total MP (Fig. 1B). Fibers of poly(ethylene terephthalate) (89.4%), polyacrylate (75.0%), polypropylene (16.0%), and polyethylene (3.95%) were observed (Table 2), collectively accounting for 22.2% of the total MP detected (Fig. 1B). These findings point to synthetic textiles as a likely source of fiber contamination, potentially introduced through filtration systems, protective clothing (such as lab coats, gloves, or hairnets in laboratory or food processing settings), remnants of synthetic materials, or airborne fibers settling during sample processing^50^. Irregular shapes were predominant across most polymers (77.4% of the total MP; Fig. 1B). The high occurrence of irregular fragments is likely due to mechanical degradation of plastic materials, potentially originating from packaging, processing equipment, or the wear and tear caused by friction between machine components and surfaces during the production process^50^. These results are consistent with those reported by Buyukunal^47^, who also found that fragments and fibers were the predominant MP shapes. However, their study on milk-based beverages revealed a different proportion in terms of shapes, with fibers representing the most abundant MP morphology (49.8%), followed by fragments (14.7%), and spheres (12.4%).

**Table 2:** Frequency of different shapes and colors of MP detected in dairy products

MP	Detected MP, *n*	Shape, %			Color, %					
		Beads	Irregular fragment	Fiber	Black	Blue	Brown	Gray	Red	Transparent
Chlorinated polyethylene	5	–	100.0	–	40.0	20.0	–	–	40.0	–
Copolymers^a^	16	6.25	93.8	–	31.3	6.25	6.25	56.3	–	–
Ethylene vinyl acetate	2	–	100.0	–	–	–	50.0	50.0	–	–
Nylon	5	–	100.0	–	20.0	–	–	80.0	–	–
Polyacrylate	8	–	25.0	75.0	25.0	25.0	25.0	25.0	–	–
Polybutadiene	1	–	100.0	–	–	–	100.0	–	–	–
Polyester	4	–	100.0	–	50.0	–	–	50.0	–	–
Polyethylene	76	–	96.1	3.95	1.32	–	13.2	84.2	1.32	–
Poly(ethylene terephthalate)	47	–	10.6	89.4	19.2	12.8	10.6	53.2	–	4.26
Polyisoprene	5	–	100.0	–	–	20.0	80.0	–	–	–
Polyoxymethylene	1	–	100.0	–	–	–	–	100.0	–	–
Polyphenylene sulfide	1	–	100.0	–	100.0	–	–	–	–	–
Polypropylene	50	–	84.0	16.0	2.00	2.00	12.0	82.0	2.00	–
Polystyrene	18	–	100.0	–	5.56	–	11.1	83.3	–	–
Polytetrafluoroethylene	5	–	100.0	–	–	–	60.0	40.0	.–	–
Polyurethane	2	–	100.0	–	50.0	–	50.0	–	–	–
Polyvinyl chloride	6	–	100.0	–	–	–	16.7	83.3	–	–
Polyvinylidene fluoride	1	–	100.0	–	–	–	–	100.0	–	–
Resin	1	–	100.0	–	–	–	–	100.0	–	–
Silicone	12	–	100.0	–	–	–	25.0	75.0	–	–

^a^Copolymers class comprises: acrylonitrile–butadiene–styrene, chlorinated polyethylene–polystyrene copolymer, polyacrylonitrile–butadiene–styrene copolymer, polyethylene–nylon copolymer, polyethylacrylate–polystyrene–polyacrylamide copolymer, polystyrene–polyacrylate copolymer, polystyrene–polybutadiene copolymer, silicone–polyisoprene copolymer, and styrene–ethylene–butylene–styrene.

The frequencies of MP color detected in dairy products are shown in Table 2. Gray MP was predominantly observed in polyethylene (84.2%), polystyrene (83.3%), polyvinyl chloride (83.3%), and polypropylene (82.0%). Brown and black MP were detected in smaller proportions, being identified in 13 and 11 out of the 20 identified polymers, respectively. Transparent MP was exclusively found in poly(ethylene terephthalate) (4.26%), which may be attributed to specific packaging components. As shown in Fig. 1C, about 93% of the MP identified in this study were pigmented, with gray (68.4%), brown (15.0%), and black (9.77%) being the most prevalent colors. However, Buyukunal^47^ reported a different color distribution, with blue (19.8%), red (17.9%), and transparent (15.0%) being the most abundant, followed by black (11.2%). Moreover, it is important to consider that various color pigments can be added to polymer mixtures during the production process. The colors observed under the microscope may therefore result from the pigments and additives used in the manufacturing of plastic packaging^46^. For this reason, Fourier-transformed infrared micro-spectroscopy confirmation is essential for a conclusive identification. The predominance of gray MP observed in our study supports the hypothesis that the primary sources of contamination in dairy products are related to food-contact plastics and packaging materials. Gray MP is commonly associated with industrial plastic components used in food packaging, such as films, liners, and closures, which often contain neutral pigments^30^. Their presence may result from mechanical abrasion, aging, or degradation of these materials during processing, handling, or storage, rather than from environmental contamination^35,41^.

### Quantification of MP in dairy products

Descriptive statistics of MP concentration in dairy products are presented in Table 3. A total of 28 dairy product samples were analyzed for MP contamination, with MP detected in 26 of them. Overall, 266 plastic particles were identified, belonging to 20 different polymer classes. Poly(ethylene terephthalate) was found in 19 samples, being the most frequently identified polymer, followed by polyethylene (found in 15 samples), polypropylene (found in 12 samples), and copolymers (found in 12 samples). Polyethylene exhibited the highest average concentration (640.0 MP/kg), followed by polypropylene (516.7 MP/kg) and poly(ethylene terephthalate) (357.9 MP/kg). Notably, some polymers were detected in isolated cases; these included polybutadiene, polyoxymethylene, polyphenylene sulfide, polyvinylidene fluoride, and resin, which were found in single samples. Silicone exhibited the greatest coefficient of variation (127.3%), suggesting significant variability in the analyzed samples. In contrast, polyacrylate, polyvinyl chloride, and polypropylene showed the lowest coefficients of variation (35.0, 38.3, and 38.6%, respectively), indicating a more homogeneous distribution across contaminated samples.

**Table 3:** Descriptive statistics of MP concentration (MP/kg) in dairy products

MP	*n* of contaminated samples	Mean	SD	CV, %	Minimum	Maximum
Chlorinated polyethylene	3/28	333.3	230.9	69.3	200.0	600.0
Copolymers^a^	12/28	283.3	232.9	82.2	200.0	1000
Ethylene vinyl acetate	2/28	200.0	–	–	–	–
Nylon	5/28	200.0	–	–	–	–
Polyacrylate	6/28	233.3	81.7	35.0	200.0	400.0
Polybutadiene	1/28	200.0	–	–	–	–
Polyester	3/28	266.7	115.5	43.3	200.0	400.0
Polyethylene	15/28	640.0	442.1	69.1	200.0	2,000
Poly(ethylene terephthalate)	19/28	357.9	171.0	47.8	200.0	800.0
Polyisoprene	3/28	333.3	230.9	69.3	200.0	600.0
Polyoxymethylene	1/28	200.0	–	–	–	–
Polyphenylene sulfide	1/28	200.0	–	–	–	–
Polypropylene	12/28	516.7	199.2	38.6	200.0	800.0
Polystyrene	9/28	311.1	202.8	65.2	200.0	800.0
Polytetrafluoroethylene	5/28	200.0	–	–	–	–
Polyurethane	2/28	200.0	–	–	–	–
Polyvinyl chloride	5/28	240.0	89.4	38.3	200.0	400.0
Polyvinylidene fluoride	1/28	200.0	–	–	–	–
Resin	1/28	200.0	–	–	–	–
Silicone	4/28	550.0	700.0	127.3	200.0	1,600

^a^Copolymers class comprises: acrylonitrile–butadiene–styrene, chlorinated polyethylene–polystyrene copolymer, polyacrylonitrile–butadiene–styrene copolymer, polyethylene–nylon copolymer, polyethylacrylate–polystyrene–polyacrylamide copolymer, polystyrene–polyacrylate copolymer, polystyrene–polybutadiene copolymer, silicone–polyisoprene copolymer, and styrene–ethylene–butylene–styrene.

*SD* standard deviation, *CV* coefficient of variation.

Similar MP concentrations have been observed in other studies. For instance, in a study on MP in Turkish milk-based beverages, Buyukunal^47^ reported MP concentrations ranging from 3 to 43 MP/100 mL, with the highest contamination levels detected in samples collected after the homogenization and pasteurization steps. The same authors reported an average concentration of 19 MP/100 mL in raw milk, suggesting that the contamination in the raw matrix plays a crucial role in determining the overall MP content in the final product. Rbaibi Zipak et al.^46^, in their investigation of MP contamination throughout the yogurt production process reported concentrations ranging from 2 to 58 MP/100 mL, with the lowest contamination found in the early stages of processing (in the bulk tank milk), and the highest contamination detected in the final stages (during the yogurt cup filling process). Da Costa Filho et al.^33^ identified MP concentrations in different types of milk, including raw milk, whole and skimmed liquid milk, and powdered milk products, ranging from 204 to 1004 MP/100 mL, with polyethylene and polypropylene being the predominant polymers, which is consistent with our findings. In contrast, Kutralam-Muniasamy^41^ and Basaran^43^ found considerably lower MP concentrations in commercial liquid milk from different brands, ranging from 1–16 MP/L to 3–11 MP/L, respectively. The large variability of MP levels detected across different studies can be attributed to differences in sample characteristics—such as type of dairy product, fat content, and origin—as well as to the specific stage of the dairy production process sampled along with the MP measurement protocols employed. Common sources of MP include plastic-based packaging, food-contact materials used during processing, and synthetic fibers from operators' clothing^30,31^.

Similar levels of contamination have been observed in other food products, particularly in meat. For instance, Visentin^51^ identified a total of 898 plastic particles in beef hamburgers, belonging to 18 different polymer classes. The detection of different polymer classes, including polycarbonate, polyethylene, and polypropylene, highlights the high variability of plastic contaminants in meat products, which is consistent with the polymer diversity observed in the present study. Kedzierski et al.^52^ demonstrated that plastic particles from polystyrene trays used in the commercial packaging of chicken meat adhered to the meat surface even after rinsing, highlighting a potential risk for consumers. Contamination pathways identified along the meat supply chain could also apply to the dairy sector, with packaging and processing equipment as possible common sources of MP contamination across different food matrices.

### Variability of MP contamination among dairy products

**Fig. 2:**
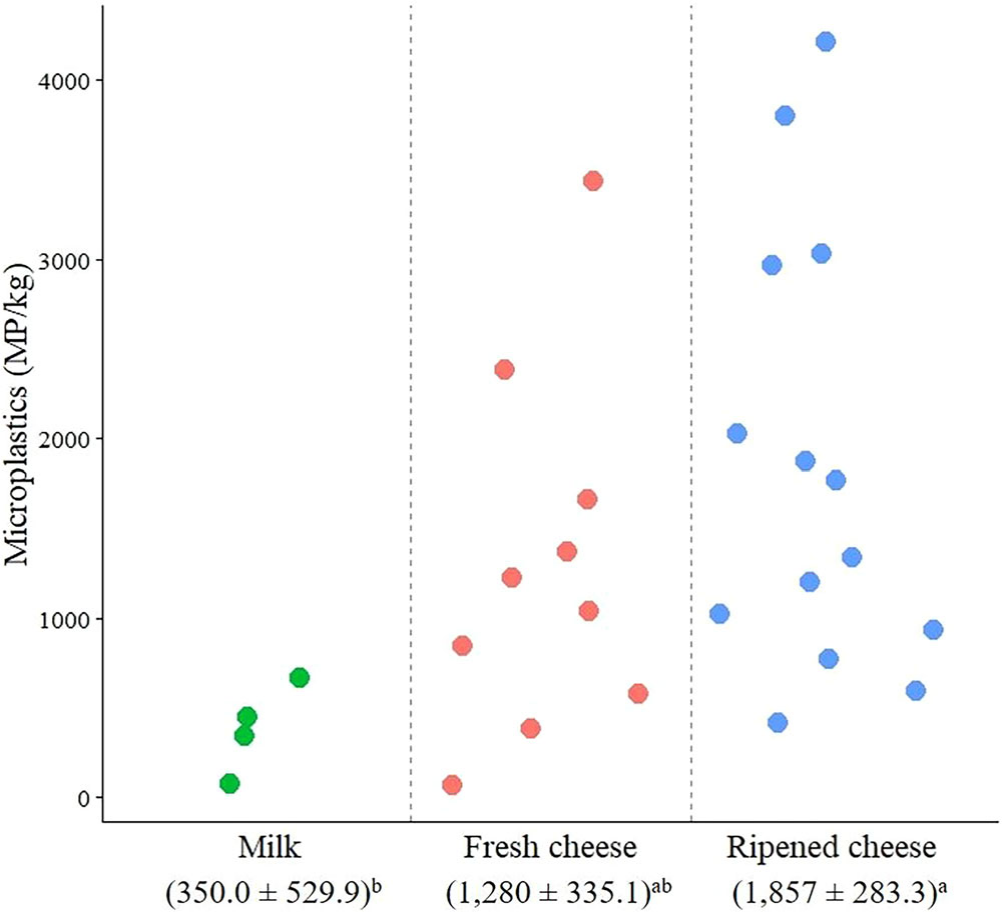
Variability of MP in dairy products. Data points and LSMs were calculated as the sum of the individual MP concentrations (MP/kg) identified in each sample, grouped into three types of dairy products. Means with different superscript letters differ significantly (*P* < 0.05).

Data points and least square means (LSMs) of the overall MP concentration detected in milk, fresh cheese, and ripened cheese samples are presented Fig. 2. The analysis of variance highlighted that ripened cheese had the highest MP concentration (1857 MP/kg), followed by fresh cheese (1280 MP/kg), and milk (350.0 MP/kg; *P* < 0.05). Current literature provides scarce information on the variability of MP contamination in dairy products, particularly in relation to different processing stages and different cheese types. Kutralam-Muniasamy et al.^41^ analyzed 23 milk samples from various brands in Mexico, including whole milk, lactose-free milk, and low-fat milk. They found that MP were present in all the analyzed samples, with processed milk (such as lactose-free and low-fat varieties) exhibiting higher concentrations of MP compared to whole milk. In a study on MP contamination in milk products, Da Costa Filho^33^ analyzed 8 samples, including 2 raw milk samples, 3 whole milk samples, 1 skim milk sample, and 2 skim milk powder samples. They found that powdered milk contained the highest concentration of MP, with contamination linked to ubiquitous environmental plastics, packaging materials, and processing (particularly the use of polymeric membranes during filtration and drying). Badwanache et al.^53^ analyzed 21 milk samples, including 7 raw milk samples directly transferred from the farm to the laboratory into steel containers, 7 milk samples from different dairy companies packed in polyethylene plastic bags, and 7 commercially branded milk samples in polyethylene plastic bottles. Their findings revealed that the commercially branded milk samples exhibited the highest MP concentration, which was primarily attributable to the plastic packaging. In contrast, milk collected directly from farms or dairies showed lower contamination levels, confirming the contribution of packaging to increased MP contamination.

In the present study, cheese was found to have higher levels of MP compared to milk. This is likely due to the cheese-making process, as a procedure that removes whey, thereby reducing total mass and concentrating solid components, including any MP fragments^41^. In other words, it is likely that MP are not preferentially removed through the whey fraction, resulting in their concentration in the curd. This phenomenon is particularly evident in ripened cheese, where moisture loss during aging and additional processing steps may further contribute to MP accumulation. To the best of the authors’ knowledge, no studies have specifically investigated MP contamination in cheese. However, Banica^48^ investigated the occurrence of MP in high-fat dairy products, including 11 butter samples from various brands (both conventional and organic), as well as 7 sour cream samples from different producers. Their findings indicated that conventional butter had the highest MP concentration (7875 MP/kg), followed by sour cream (5600 MP/kg), and organic butter (2500 MP/kg). These findings are in line with the results of the present study, where dairy products undergoing more intensive processing and containing higher fat levels—such as cheese—were found to have higher MP concentrations than less processed low-fat products such as liquid milk. This supports the hypothesis that intensive processing and high-fat content may facilitate MP accumulation.

Moreover, the consistent detection of MP across different products suggests that multiple contamination sources – introduced at various stages of the production chain – likely contribute to the differences in MP concentrations observed among dairy products. Cheese making involves multiple steps, such as curd cutting, molding, pressing, and aging, all of which can increase MP exposure due to prolonged contact with processing surfaces, equipment, and food-contact materials^54^. For example, plastic molds, storage containers, and packaging films commonly used during cheese production and maturation could represent potential sources of MP contamination. The prolonged maturation time of ripened cheese could further contribute to MP accumulation, as extended storage in plastic materials may lead to the gradual release of MP particles. The differences in MP contamination levels among the three product categories analyzed in this study—liquid milk, fresh cheese, and ripened cheese—suggest that ripened cheeses are more susceptible to MP contamination, likely due to the complexity of their production processes and the materials used throughout their supply chains.

It can be concluded that MP contamination varies considerably among different dairy products. The 28 analyzed samples, including liquid milk, fresh cheese, and ripened cheese, highlighted a wide variability of MP in terms of size, shape, and color. Detected MP ranged from 24.0 to 4817 μm in size, with an average of 243.7 μm. Irregular fragments were the predominant morphology (77.4%), and the vast majority of MP were pigmented, with gray (68.4%), brown (15.0%), and black (9.77%) being the most frequent colors. The most frequently detected polymers were poly(ethylene terephthalate), polyethylene, and polypropylene, found in 19, 15, and 12 samples, respectively. Significant differences in MP concentrations were observed across dairy products, with ripened cheese exhibiting the highest MP levels (1857 MP/kg), followed by fresh cheese (1280 MP/kg) and milk (350.0 MP/kg). These findings emphasize the importance of considering the specific dairy product type, when assessing the extent of MP contamination in the dairy sector. Both the production process and the packaging materials used may influence the level of contamination, and specific mitigation strategies should be developed accordingly. Future research should be conducted at the plant level, encompassing all stages of the production process to identify specific sources of MP contamination and to support the development of effective mitigation strategies.

## Methods

### Sample collection

A total of 28 samples were purchased from large-scale retail markets, including 4 ultra-high temperature milk samples (1 L each), 10 fresh cheese samples (500 g each; less than 1 month of aging), and 14 ripened cheese samples (500 g each; more than 4 months of aging). Commercial names are not disclosed to maintain objectivity and avoid potential conflicts of interest.

After purchase, samples were transported to the laboratory of the European Center for the Sustainable Impact of Nanotechnology (ECSIN, Padova, Italy), as part of Mérieux NutriSciences Company (Chicago, USA), where they were frozen upon arrival.

### Reagents and equipment

Sample pretreatment, including quartering, digestion, microfiltration, and MP detection, was conducted in a dedicated clean room adhering to ISO 14644-1:2015 Class 7 standards (ISO, 2015).

All solutions were micro-filtered through a silver membrane filter (3.0 µm pore size, 25 mm diameter; Sterlitech Corporation, Auburn, United States). Ultrapure water (resistivity: 18.3 MΩ/cm at 25 °C) was generated using a Zeneer Power III system (Human Corporation, Garak-ro, Republic of Korea). Work surfaces were thoroughly cleaned prior and during experimental procedures to avoid sample contamination. Glassware was washed in five cycles with distilled water, followed by five rinses with ultrapure water, and oven-dried before use.

The laboratory environment was carefully managed to prevent contamination, maintaining strict control over airborne particulates, temperature, and humidity. The laboratory was equipped with a cleanroom environment with a controlled ISO class 7 standard, ensuring that High-Efficiency Particulate Air (HEPA) filters were used to maintain air quality, and positive pressure was maintained to prevent the ingress of contaminants from outside the cleanroom. Operators wore antistatic lab coats and avoided using materials, such a latex or nitrile gloves, that could release particles or residues potentially interfering with MP analyses. Hair covers were also excluded to further minimize contamination risks. To protect equipment and maintain sample purity, all tools used during sample preparation were covered with aluminum foil.

### Sample quartering, digestion, and microfiltration

Before analysis, milk samples were thawed overnight at room temperature and gently inverted five times to promote fat and solids homogenization. Therefore, an aliquot of 100 mL was used for the subsequent analytical phases. Fresh and ripened cheeses were thawed overnight, and removed from the packaging. Afterwards, a quartering step was performed to obtain a representative cheese aliquot. This procedure involved dividing each sample into four equal parts, discarding two quarters, and recombining the remaining portions. The process was repeated by further quartering the recombined portion, until a final sample weight of 10 g was achieved.

To remove organic components, a digestion protocol based on the methods described by Dehaut^44^ and EFSA^1^ was employed, involving both enzymatic and chemical digestion steps. As for the enzymatic digestion protocol, 100 mL of ultrapure water and 20 mL of 5% trypsin solution (Merck KGaA, Darmstadt, Germany) were added to 100 mL of milk and 10 g of cheese in a glass flask. The mixture was stirred at 37 °C overnight using a Shake’n Bator (EuroClone, Milan, Italy). As for the chemical digestion, 100 mL of 10% potassium hydroxide (KOH) solution was added to each sample. The mixture was stirred at 60 °C overnight in the Shake’n Bator. Finally, 100 mL of 10% ethylenediaminetetraacetic acid (EDTA) solution was added, and the mixture was stirred at 60 °C for 2 h in the Shake’n Bator.

After digestion, all samples were micro-filtered through silver membrane filters (3.0 µm pore size; Sterlitech Corporation, Auburn, United States). Density separation of MP in milk and cheese samples was performed using an oversaturated NaCl solution (Merck KGaA, Darmstadt, Germany) as flotation solution, which enabled to suspension also heavy MP fragments. This step was repeated three times to improve the separation efficiency. Throughout the microfiltration step, the filtering funnel was covered with aluminum foil to minimize contamination. Finally, the filter membrane was dried in an oven at 70 °C for 24 h.

### Identification and quantification of MP through µ-FTIR-ATR

**Fig. 3:**
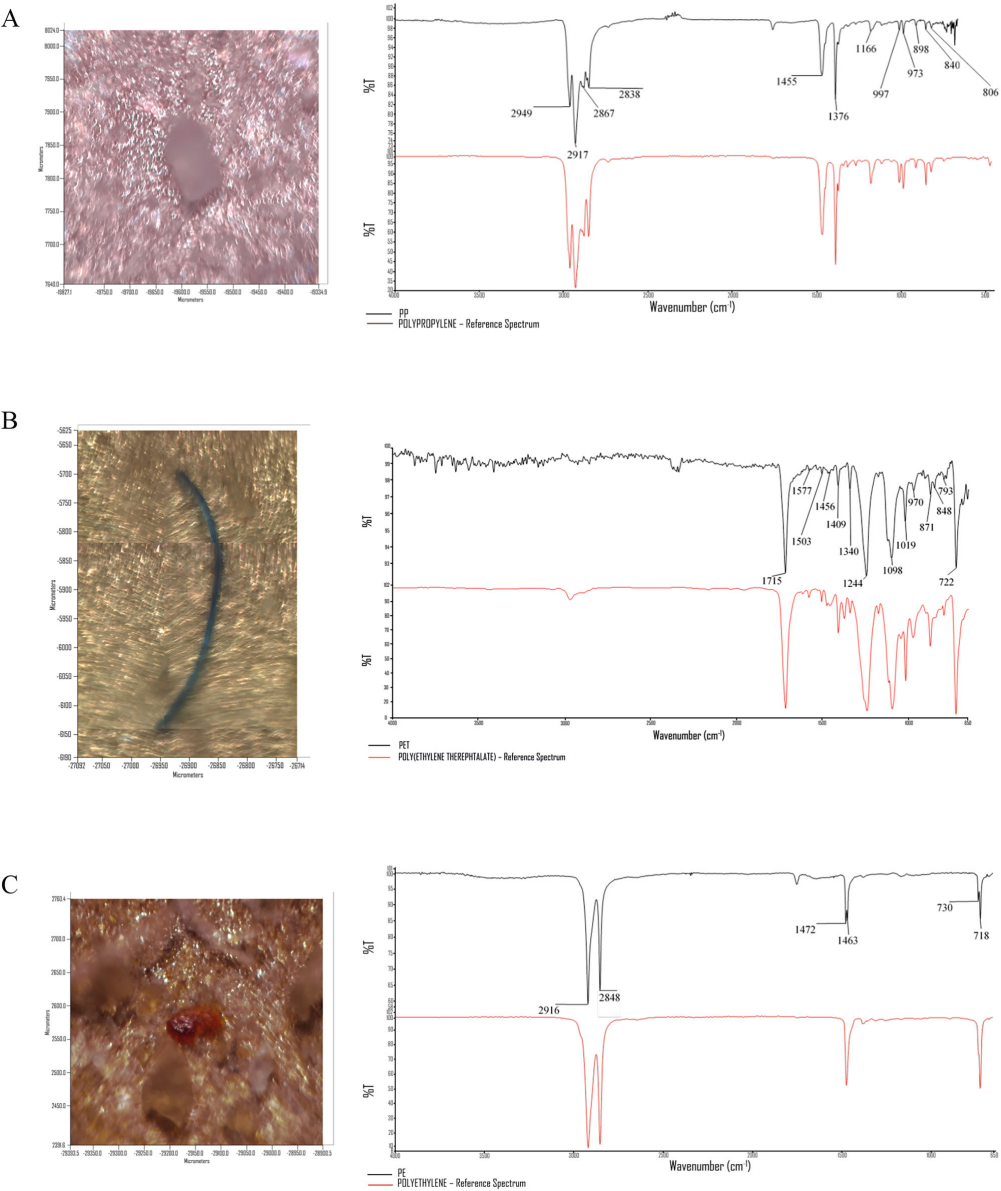
Identification of MP using μ-FTIR-ATR spectroscopy. Images and Fourier-transformed infrared micro-spectroscopy in attenuated total reflectance mode analysis (μ-FTIR-ATR) spectra of three identified MP with different shapes and colors: **A** polypropylene, **B** poly(ethylene terephthalate), and **C** polyethylene (black line for MP spectra from dairy products, red line for MP spectra from reference libraries).

Filter membranes were analyzed using an FTIR Spectrometer Frontier coupled to a microscope Spotlight 400 (PerkinElmer Italia Spa, Milan, Italy). Polymers were identified via Fourier-transformed infrared micro-spectroscopy, with spectral detection wavelengths in the range between 4000 cm^−1^ and 650 cm^−1^. Each detected MP particle underwent four repeated µ-FTIR-ATR scans, until the final average spectrum was acquired. Background spectra were recorded using air as the reference medium. To minimize errors, spectra obtained for each MP particles were corrected by subtracting the corresponding air background spectra. Therefore, spectra were compared against a reference library using Spectrum 10 software (PerkinElmer Italia Spa, Milan, Italy) (Fig. 3). Polymers were considered as correctly determined when the matching was greater than 80%. The most significant peaks are listed in Table 4, together with the associated vibrational modes.

**Table 4:** Characteristics bands with assigned vibrational modes from μ-FTIR-ATR spectra of polypropylene^55^, poly(ethylene terephthalate)^56^, and polyethylene^57^

Wavenumber (cm^−1^)	Vibrational mode
Polypropylene	
~2949	CH_3_ asymmetric stretching
~2917	CH_2_ asymmetric stretching
~2867	CH_3_ symmetric stretching
~2838	CH_2_ symmetric stretching
~1455	CH_3_ asymmetric bending
~1376	CH_3_ symmetric bending
~1166	CH_2_ twisting and CH wagging
~997	CH bending and wagging, CH_3_ rocking
~973	CH_3_ rocking, CH_2_ wagging, and CH bending
~898	C–C chain symmetric stretching
~840	CH_2_ rocking and C-CH_3_ stretching
~806	C–C chain symmetric stretching and CH_2_ rocking
Poly(ethylene terephthalate)	
~1715	Stretching vibration of the carbonyl ester group C=O
~1577, ~1503	Vibrations aromatic skeleton with stretching C=C
~1456, ~1409, ~1340	Stretching of the C–O group, deformation of the O–H group, and bending and wagging vibrational modes of the ethylene glycol segment
~1244	Terephthalate group (OOCC_6_H_4_–COO)
~1098, ~1019	CH_2_ group and vibrations of the ester C–O bond
~970, ~871, ~848	Aromatic rings 1, 2, 4, 5; Tetra replaced
~793	Vibrations of the adjacent two aromatic H in p-substituted compounds and aromatic bands
~722	Out-of-plane vibration of the benzene ring
Polyethylene	
~2916	CH_2_ asymmetric stretching
~2848	CH_2_ symmetric stretching
~1472, ~1463	CH_2_ symmetric bending
~730, ~718	CH_2_ bending and rocking

High-resolution images of MP were electronically stored using Spectrum 10 software. The size, color, and shape of each MP particle were determined through ImageJ software (ImageJ 2022). Size was measured on the maximum length of each MP particle.

### Blanks and method recovery

To assess potential contamination arising from the analytical procedure, blanks were prepared simultaneously along with each sample, following the same sample pretreatment procedures (i.e., digestion and microfiltration) and µ-FTIR-ATR analysis. Blanks were deemed acceptable if they contained no more than 10 MP fragments. If a blank exceeded this threshold (i.e., ≥11 MP fragments), the analysis of the corresponding sample was considered invalid and repeated. The number of MP contaminants detected in blanks was subtracted from the total MP fragments detected in the corresponding sample.

The recovery rate of MP was evaluated by spiking ultrapure water samples with a commercial standard of polystyrene (Cospheric LLC, Santa Barbara, CA). Spiked samples were processed following the entire digestion and filtration procedures. Ten spiking levels, ranging from 22 MP/kg to 207 MP/kg of water, were tested. Recovery rates ranged from 66% to 122%, with an average recovery of 84%.

### Statistical analyses

For each sample, the total MP concentration (MP/kg) was calculated as the sum of the concentrations of all MP classes. The total MP concentration was normally distributed, and no outliers were detected. Data were then analyzed through R Software (v. 4.1.3), according to the following linear model described in Eq. (1):

1 $${y}_{i}=\mu +\mathrm{dairy}\,{\mathrm{product}}_{i}+e$$

were *y*_*i*_ is the dependent variable (i.e., the total MP concentration detected in each sample); *μ* is the overall intercept of the model; dairy product_*i*_ is the fixed effect of the *ith* type of dairy product (three classes: milk, fresh cheese, and ripened cheese); and *e* is the random error. Multiple comparisons of LSMs were performed using the Bonferroni adjustment, with the significance threshold set at *P* ≤ 0.05.

## Data Availability

Data is provided within the manuscript.
